# Real-world pharmacovigilance investigation of imipenem/cilastatin: signal detection using the FDA Adverse Event Reporting System (FAERS) database

**DOI:** 10.3389/fphar.2025.1524159

**Published:** 2025-02-13

**Authors:** Peng Jia, Yusen Zhou, Yuan Gao, Shangyu Wang, Jiangliu Yin, Yixiang Lian, Quanyou Lai

**Affiliations:** ^1^ Department of Hepato-Biliary and Pancreato-Splenic Surgery, Xijing Hospital, Air Force Military Medical University, Xi’an, China; ^2^ Department of Neurosurgery, The Affiliated Changsha Central Hospital, University of South China, Changsha, China; ^3^ Department of Pathology, The Affiliated Changsha Central Hospital, University of South China, Changsha, China

**Keywords:** imipenem/cilastatin, FAERS, pharmacovigilance, real-world data analysis, adverse events, carbapenem

## Abstract

**Background:**

Although imipenem/cilastatin (IMI/CIL) has demonstrated favorable therapeutic efficacy against various infections, the incidence of potential adverse events (AEs) has escalated in parallel with its increased utilization and has been documented in clinical trials. However, a comprehensive understanding of real-world implications remains lacking.

**Methods:**

By conducting a comprehensive search in the FDA Adverse Event Reporting System (FAERS) database, AE reports associated with IMI/CIL as the primary suspect (PS) were selected for analysis, spanning from the first quarter of 2004 to the fourth quarter of 2023. Utilizing disproportionality analysis techniques, potential signals of AE s were identified through reported odds ratio (ROR), proportional report ratio (PRR), Bayesian confidence propagation neural network (BCPNN), and empirical Bayesian geometric mean (EBGM). The obtained results were systematically classified using Medical Dictionary for Regulatory Activities (MedDRA).

**Result:**

From the first quarter of 2004 to the fourth quarter of 2023, a total of 2,574 reports documenting AEs associated with IMI/CIL were obtained, with more than half (n = 1,517, 58.94%) involving individuals aged over 60 years old. Descriptive analysis was conducted based on age groups and time to onset, revealing that the majority of AEs occurred within 3 days. Adverse drug reactions caused by IMI/CIL were classified into 24 system organ classes (SOCs) at the preferred term (PT) level. Furthermore, previously unreported and clinically significant AEs such as cerebral atrophy, and delirium were also identified at the PT level.

**Conclusion:**

This study offers a more comprehensive insight into the monitoring, supervision, and management of adverse drug reactions associated with IMI/CIL. Clinicians should pay further attention to the implications of numerous AEs and their corresponding signal intensities, as well as unrecorded signals of severe AEs. This holds significant value in enhancing the clinical safety profile of IMI/CIL.

## 1 Introduction

To address the issue of limited antimicrobial options due to resistance to β-lactam drugs, an atypical β-lactam antibiotic that is stable against β-lactamase, namely, carbapenems, has been identified (Bush and Bradford, 2020; Drawz and Bonomo, 2010). Carbapenems exhibit stability against the majority of β-lactamases, including AmpC, β-lactamases, and extended-spectrum β-lactamases (ESBL), and can serve as a feasible therapeutic alternative to third-generation cephalosporins (Zhanel et al., 2007; Bradley et al., 1999). Since the mid-1980s, carbapenems have been extensively utilized in clinical practice and are regarded as the preferred drugs for multi-drug-resistant infections. Imipenem (IMI) is the first carbapenem antibiotic to be approved (Paterson and Bonomo, 2005; Paterson, 2000). Compared with other β-lactam drugs, it possesses a broader spectrum of antibacterial activity and post-antibiotic effect (PAE) against both Gram-positive and Gram-negative bacteria (MacKenzie et al., 1994). IMI is metabolized by dehydropeptidase-1 (DHP-1) produced by proximal renal tubules and is rapidly inactivated. Cilastatin (CIL), a DHP-1 inhibitor, were formulated in a fixed 1:1 ratio to delay the metabolism of imipenem *in vivo* and prevent the inherent nephrotoxicity of imipenem. Clinical experience has demonstrated that imipenem/cilastatin (IMI/CIL) demonstrates excellent efficacy in the treatment of moderate to severe infections in various systems and is frequently employed as empirical treatment for febrile neutropenia and other serious infections caused by multi-drug resistant pathogens (Balfour et al., 1996).

In the 1980s, Calandra et al. conducted a comparative analysis of the efficacy and safety of IMI/CIL and cephalosporins in two multicenter clinical trials conducted in North America and Europe. The results indicated that the safety of IMI/CIL was comparable to that of conventional β-lactam antibiotics (Calandra et al., 1983). Besides, Calandra et al. also conducted a review of case reports involving approximately 3,500 patients following the use of IMI/CIL, and the data indicated that common adverse effects (AEs) included local reactions at the site of intravenous infusion (such as phlebitis and local pain), gastrointestinal symptoms (including nausea, vomiting, and diarrhea), impaired liver function, elevated eosinophils, skin symptoms (such as rash, pruritus, and urticaria), seizures, oral mucosal changes, fever, and dizziness (Calandra et al., 1988a). Additionally, rare AEs associated with IMI/CIL included severe neutropenia (Fariñas et al., 1993), thrombocytopenia (Qiao et al., 2022), persistent hiccups unrelated to central nervous system diseases (Lucena et al., 1992), and exacerbation of myasthenia gravis (O’Riordan et al., 1994). Some experiments have demonstrated the potential of IMI/CIL to induce nephrotoxicity (Tahri et al., 2018). The potential toxic effects of high doses of IMI/CIL on the gonads and male reproductive organs require further investigation (Tahri et al., 2017; Cherif et al., 2019). As a classic carbapenem, IMI/CIL continues to hold significant value in contemporary clinical practice. However, the escalating utilization of IMI/CIL has brought to light potential AEs. Although Ge et al. performed an overall pharmacovigilance analysis of carbapenems using the FARES database, a comprehensive understanding of the real-world safety profile of IMI/CIL has not been achievable due to the long history and the small number of included reports (Ge et al., 2021). Not only that, over the past decade, no study has been capable of systematically summarizing its AEs, and the existing studies are merely confined to a single symptom of AEs, which severely restricts clinicians’ ability to identify and respond to other related AEs.

The US Food and Drug Administration (FDA) Adverse Event Reporting System (FAERS) database, the largest global repository of spontaneously reported AEs, systematically collects and monitors AEs associated with a wide range of approved drugs from manufacturers, healthcare professionals, and members of the public. This study comprehensively evaluates the potential risks of IMI/CIL in clinical application through signal mining of the FAERS database, providing valuable insights into its safety profile for clinical practice.

## 2 Materials and methods

### 2.1 Data sources

Since 2004, the FAERS database (https://fis.fda.gov/extensions/FPD-QDE-FAERS/FPD-QDE-FAERS.html), supervised by the FDA, has remained consistently accessible to the public, amassing a substantial volume of AE reports from real-world populations and evolving into a pivotal post-marketing safety monitoring database. This study encompasses data on reported adverse events associated with IMI/CIL spanning from Q1 2004 to Q4 2023. To ensure precision and consistency, SAS and MYSQL software were employed for data preprocessing. Individual Safety Reports (ISRs) were utilized to eliminate duplicate entries.

### 2.2 Standardization of drug names and adverse drug reactions

The American Standard Code for Information Interchange (ASCII) was extracted from the FAERS database and imported into SAS and MYSQL software for data cleansing. AE reports with IMI/CIL as the primary suspect (PS) were meticulously sorted. The RxNorm standard was utilized to accurately encode drug names, followed by the use of the latest MedDRA (25.0) to precisely match the preferred term (PT) of IMI/CIL-related AEs and categorize them into corresponding system organ classes (SOCs), thus establishing a comprehensive framework for ADR analysis. Basic clinical information of patients in the AE reports, including age, sex, country, reporter, time to onset, and outcomes of AEs such as hospitalization, death, life-threatening and disability were obtained to comprehensively evaluate drug safety. This auxiliary information provides a thorough understanding of IMI/CIL’s safety profile and potential risks in real-world settings as well as regional and national usage characteristics.

### 2.3 Signal mining algorithm

The analysis of disproportionality holds significant value in pharmacovigilance research. In this study, we employed four distinct algorithms simultaneously to meticulously identify signals of AEs associated with IMI/CIL. These algorithms encompass reported odds ratio (ROR), Proportional report ratio (PRR), Bayesian Confidence propagation Neural Network (BCPNN), and empirical Bayesian geometric mean (EBGM). The specific algorithm utilized aligns with previous research findings (Zhang et al., 2024). Utilizing four methods for the identification and quantification of AE signals can substantially enhance result stability and diminish the occurrence of false positive signals. To ensure the precision of ADR study results, the criteria for detecting positive safety signals were set as follows: ROR ≥ 3 (95% CI > 1), PRR ≥ 2 (95% CI > 1), IC025 > 1, and EBGM05 > 2.

## 3 Result

### 3.1 The basic clinical characteristics of IMI/CIL-related AEs

**FIGURE 1:**
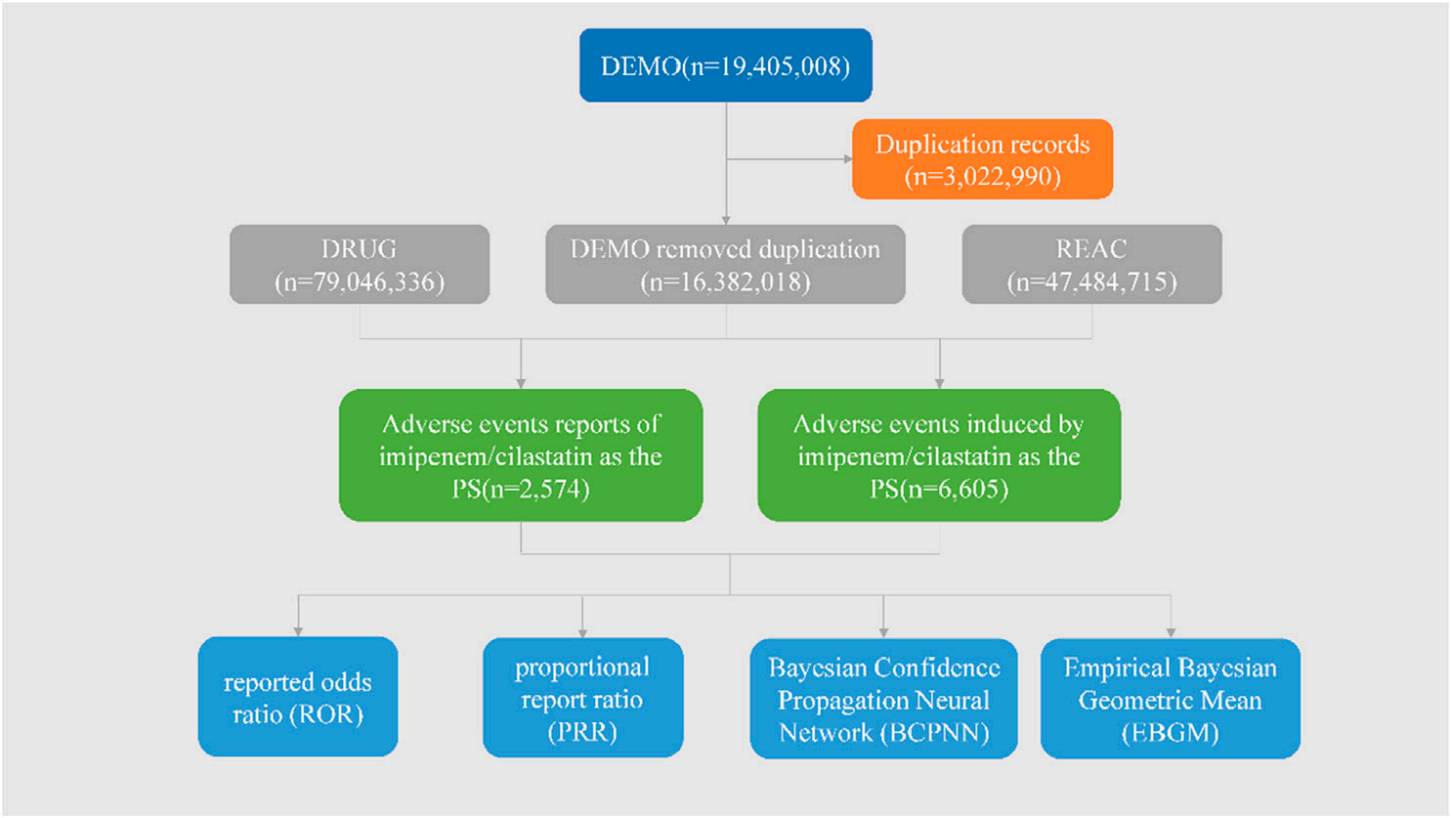
The flow diagram of selecting IMI/CIL-related AEs from FAERS database.

**FIGURE 2:**
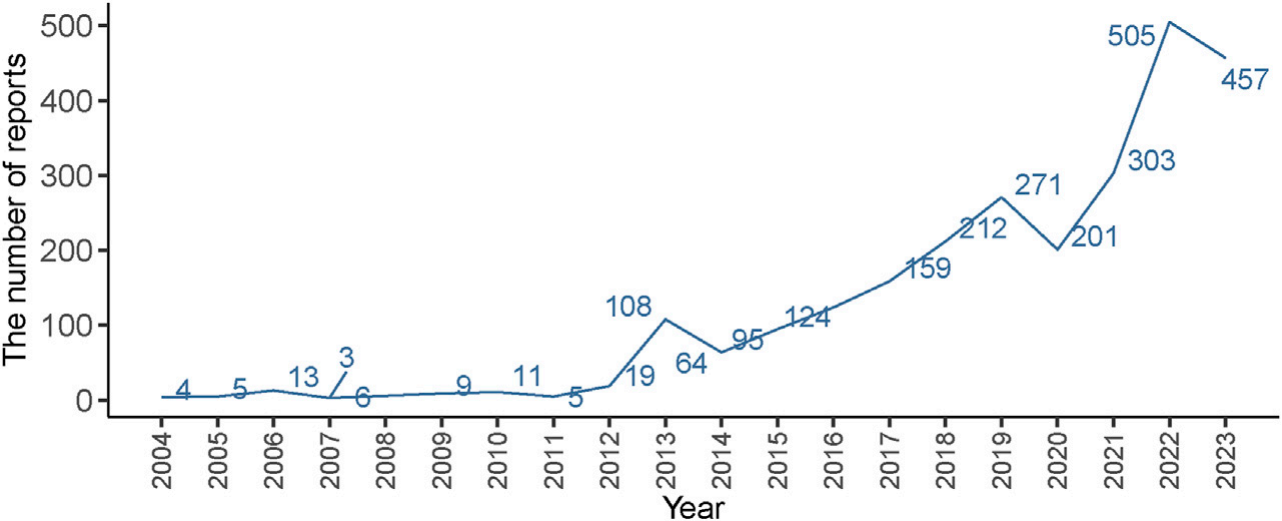
Distribution of AEs of IMI/CIL from 2004 Q1 to 2023 Q4.

In this study, a total of 19, 405, 008 ADR reports related to IMI/CIL from 2004Q1-2023Q4 were retrieved, resulting in 16, 382, 018 unique reports after the removal of 3,022,990 duplicates. Among these reports, IMI/CIL was identified as the PS drug in 2,574 reports which encompassed a total of 6,605 AEs (Figure 1). The annual line chart illustrates a gradual increase in ADR reports over time (Figure 2). Table 1 presents detailed clinical characteristics associated with reports of ADR. In the ADR reports, there was a significantly higher proportion of male patients (n = 1,490, 57.89%) compared to female patients (n = 1,026, 39.86%). More than half of the reports were over 60 years old (n = 1,517, 58.94%), which were more likely to experience AEs due to weaker organ function and increased underlying diseases. The majority of ADR reported for IMI/CIL were from pharmacists (n = 1,422, 55.24%), physician (n = 268, 10.41%), and other health-professional (n = 716, 27.82%), with only a small number from consumers (n = 160, 6.22%). Among the reporting countries, the majority of ADR reports originated from China (n = 17,644, 55.24%), followed by France (n = 272, 10.57%) and Japan (n = 106, 4.12%). Serious consequences accounted for more than 90% of the AEs reported; among these other serious was most common (n = 1984, 60.14%), followed by hospitalization (n = 823, 24.95%), life threatening (n = 209, 6.34%), death (n = 207, 6.27%), disability (n = 62, 1.88%).

**TABLE 1:** Basic information on AEs related to IMI/CIL.

Characteristics	Counts (n)	Proportion (%)
Number of events	2,574	
Gender		
Female	1,026	39.86%
Male	1,490	57.89%
Unknown	58	2.25%
Age		
<20	98	3.81%
20–40	265	10.30%
40–60	527	20.47%
≥60	1,517	58.94%
Unknow	167	6.49%
Reporter		
Pharmacist	1,422	55.24%
Other health-professional	716	27.82%
Physician	268	10.41%
Consumer	160	6.22%
Unknown	7	0.27%
Lawyer	1	0.04%
Reported countries		
China	1764	68.53%
Other	432	16.78%
France	272	10.57%
Japan	106	4.12%
Route		
Intravenous drip	1,547	60.10%
Intravenous	541	21.02%
Other	460	17.87%
Parenteral	15	0.58%
Intraperitoneal	11	0.43%
Outcomes		
Other serious	1984	60.14%
Hospitalization	823	24.95%
Life threatening	209	6.34%
Death	207	6.27%
Disability	62	1.88%
Required intervention to Prevent Permanent Impairment/Damage	12	0.36%
Congenital anomaly	2	0.06%

### 3.2 Time to onset and gender distribution analysis

**FIGURE 3:**
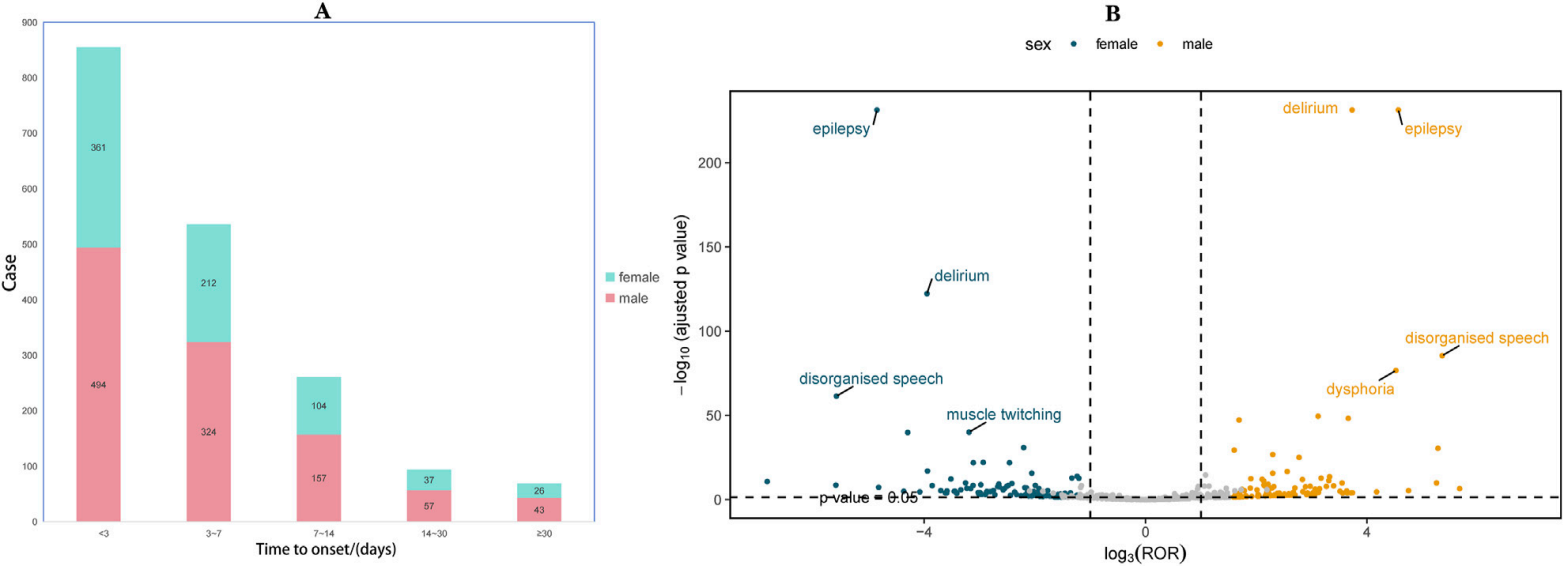
Time to onset and gender distribution of IMI/CIL-related AEs. **(A)** Result of time to onset; **(B)** Gender-differentiated risk signal volcano plot for IMI/CIL. The horizontal coordinate shows the log2 ROR value and the vertical coordinate indicates the adjusted p-value after -log10 conversion. Significant signals are highlighted and annotated in prominent colors. The p-value is adjusted with false discovery rate (FDR) method.

To further investigate the association between ADR and time, we categorized the occurrence of ADR into intervals of less than 3 days, three to 7 days, one to 2 weeks, 2 weeks to 1 month, and more than 1 month. As depicted in the pyramid diagram (Figure 3A), it is evident that ADRs predominantly manifest within the initial 3 days following medication initiation, with a majority occurring within the first week. However, the prolonged time interval between the occurrence of certain ADRs and the initiation of initial medication also warrants careful attention and vigilance. Moreover, we conducted a stratified analysis of IMI/CIL-related AE signals based on gender and generated a volcano plot. Each point within the plot represents an AE signal, and statistically significant signals were labelled. In male individuals, the most salient signals were epilepsy, delirium, disorganized speech, and muscle twitching; whereas in female individuals, the most prominent signals were epilepsy, delirium, disorganized speech, and dysphoria (Figure 3B).

### 3.3 Signal mining at the SOC level

**FIGURE 4:**
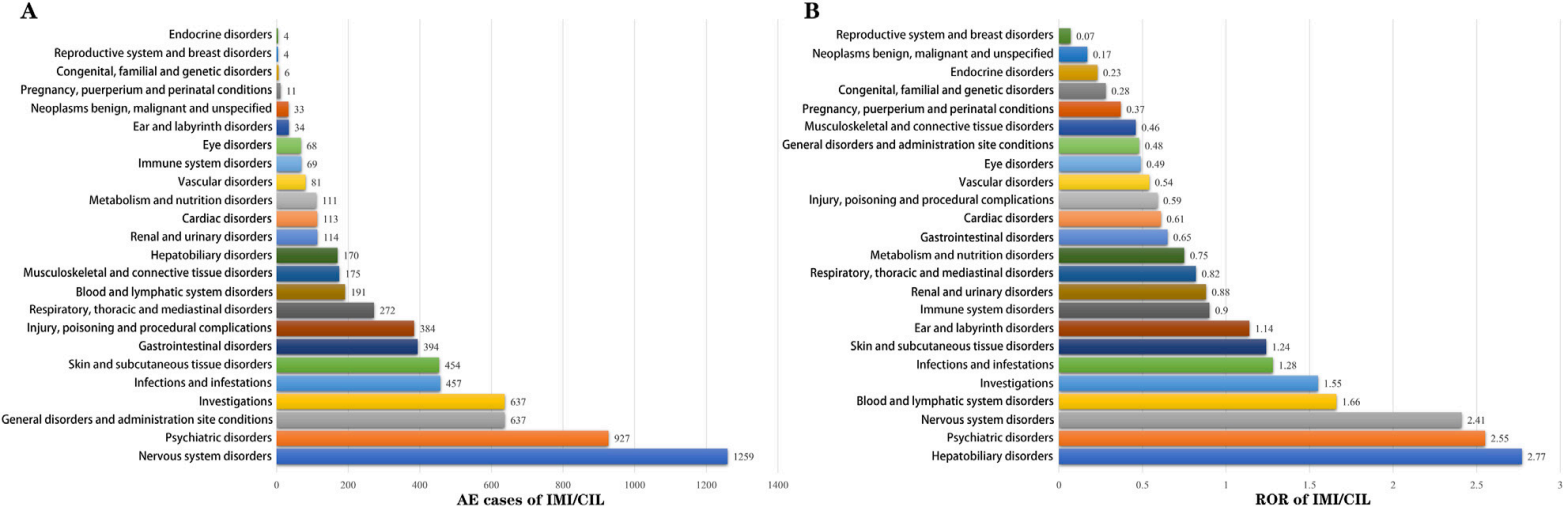
The bar chart displays the AEs at each SOC level. **(A)** Rank by AE cases; **(B)** Rank by ROR.

Figure 4; Supplementary Table S1 presents the mining results of AE signals associated with IMI/CIL in the FAERS database at the SOC level. A total of 24 SOCs were identified as being linked to the ADR. When ranked by number of cases, the top three SOCs are as follows: nervous system disorders (n = 1,259, ROR = 2.15, PRR = 2.15, IC = 1.1, EBGM = 2.14), psychiatric disorders (n = 927, ROR = 2.55, PRR = 2.33, IC = 1.22, EBGM = 2.33) and investigations (n = 637, ROR = 1.55, PRR = 1.5, IC = 0.58, EBGM = 1.5). While neurological/psychiatric disorders have been reported in drug instructions, our findings suggest that their severity may not be consistent with previous reports; it appears that IMI/CIL may exhibit greater neurotoxicity compared to other β-lactam antibiotics.

### 3.4 Signal mining at the PT level

**FIGURE 5:**
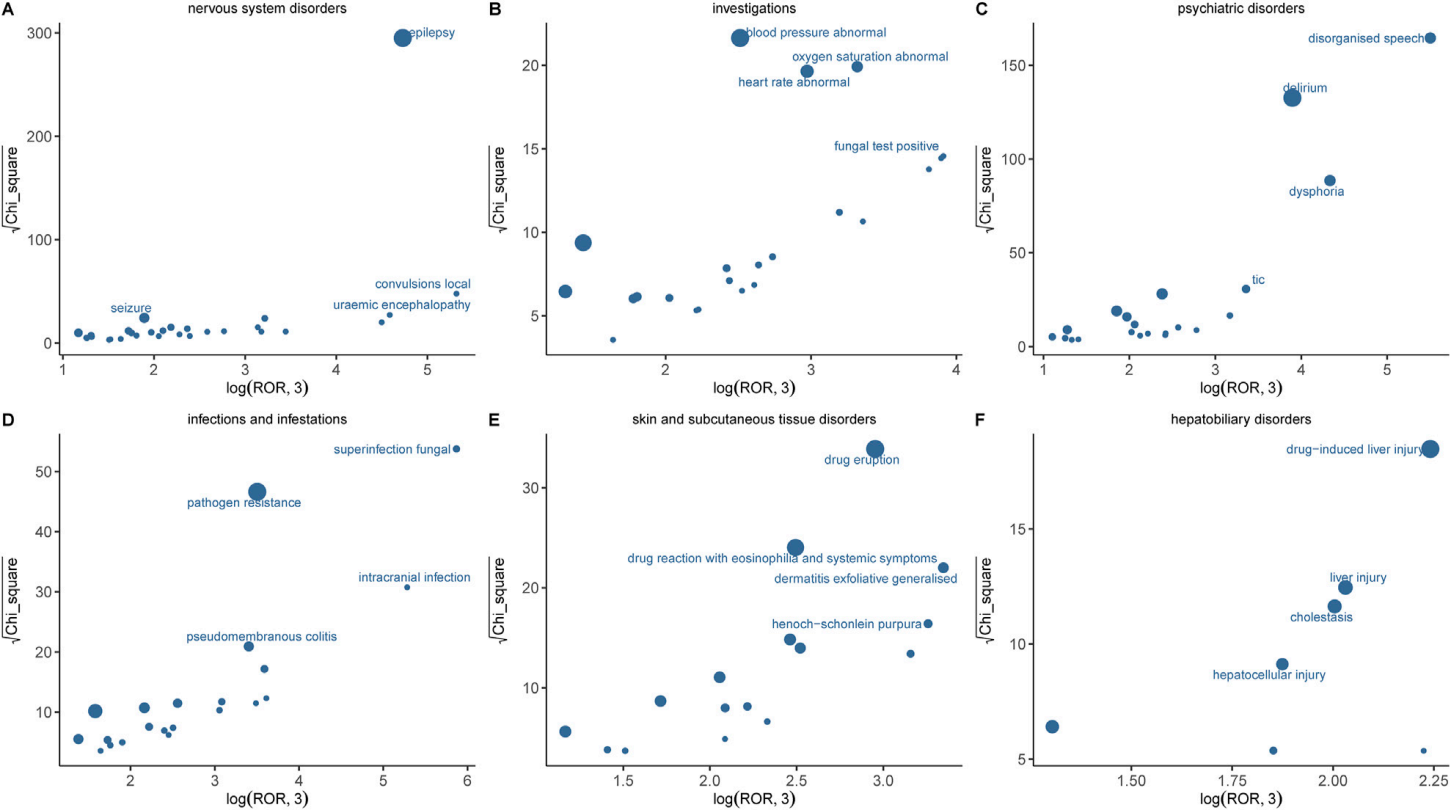
Comparison of six SOCs safety signals of IMI/CIL. **(A–F)** respectively presents the mining outcomes of adverse event signals of IMI/CIL in six system organ classes. The x-axis represents log2ROR, and the y-axis represents the square root of the χ2 value. In this graph, each point indicates a mined AE signal, with the size of the point corresponding to the quantity of reported AEs. The location of each AE in the graph is determined by the ROR (Reporting Odds Ratio) and PRR (Proportional Reporting Ratio) methods. If a point is located higher and further to the right in the graph, both algorithms suggest that the signal for that AE is strong.

Supplementary Table S2 presents the PT detection results sorted by ROR value, identifying a total of 100 ADR terms across 16 SOCs in this study. In addition, the six systems with significant signals encompassed nervous system disorders, psychiatric disorders, hepatobiliary disorders, investigations, infections and infestations, as well as skin and subcutaneous tissue disorders. Results with significant signals were visualized based on the ROR and chi-square values of AE (Figure 5). The most significant AE signal was observed within the Infections and infestations level, such as superinfection fungal (n = 5, ROR = 631.02). Epilepsy (n = 544, ROR = 180.64) emerged as the predominant AE signal among nervous system disorders, which consistent with the drug instruction recorded. Notably, toxic encephalopathy (n = 18, ROR = 34.22), cerebral atrophy (n = 8, ROR = 17.11), language disorder (n = 8, ROR = 31.48) were not previously documented in drug inserts or clinical trials. Within psychiatric disorders, disorganised speech (n = 69, ROR = 421.54), dysphoria (n = 70, ROR = 116.9) and delirium (n = 263, ROR = 72.31) exhibited the highest signal intensities and quantity but were never recorded in drug inserts. At the level of Musculoskeletal and connective tissue disorders, muscle twitching (n = 84, ROR = 31.64, 95%CI = 25.5–39.26) emerged as a significant AE signal with a substantial number of occurrences, consistent with the information provided in the drug label and thereby enhancing the credibility of our study. Other findings also demonstrating high intensity AE signals include dysbiosis, uremic encephalopathy, irregular sleep wake rhythm disorder and halo vision; their potential association with IMI/CIL warrants further investigation through larger clinical trials and fundamental research.

## 4 Discussion

In this study, we conducted a pioneering and comprehensive analysis of AE signals associated with IMI/CIL using various data analysis methods based on real-world AE reports. We identified 2574 AE reports where IMI/CIL was the PS drug. Our study offers extensive insights into the AEs linked to IMI/CIL, confirming existing safety concerns while also uncovering previously unreported AEs. The findings of this research validate that the major AEs of IMI/CIL are concentrated in six systems: nervous system disorders, psychiatric disorders, hepatobiliary disorders, investigations, infections and infestations, and skin and subcutaneous tissue disorders. A considerable proportion of these AEs were not referred to in previous studies. Furthermore, the research results indicate that AEs primarily occur in men, mainly in the elderly population (Age ≥ 60), and the occurrence of AEs is concentrated within the first 3 days after medication administration. The subsequent content presents a detailed discussion of our research result.

The self-reported AE reporting system exhibits a partial amount of missing or unknown data due to the variations in reporting subjects, which can be attributed to the non-mandatory nature of reporting. Regarding gender distribution, there is a significantly higher number of male (57.89%) reports compared to female (39.86%) reports, potentially influenced by the larger proportion of male patients with severe infections. This observation is in accordance with the outcomes of the RESTORE-IMI 2 Study (Titov et al., 2021). However, considering the intricate etiology within the spectrum of infectious diseases, this may impede our ability to observe and establish a clear association between gender and IMI/CIL-related AEs. Indeed, when evaluating drug safety, the analysis of gender differences must be taken into consideration, which is conducive to more precise management of AE. Our analysis of gender differences reveals that, in IMI/CIL-related AEs, the prevalent AEs in both men and women are generally consistent, mainly manifested as nervous system and psychiatric disorders, such as epilepsy, delirium, and disorganized speech. Nevertheless, it is notable that men are more prone to muscle twitching, which might be ascribed to the lesser muscle mass in women, leading to less obvious or less frequent muscle twitching. The research conducted by Abraham et al. indicates that in the ultrasonic diagnosis of neuromuscular diseases, a lower muscle thickness is often associated with false-negative outcomes (Abraham et al., 2023). In terms of age, AE primarily affects individuals aged 60 and above (58.94%), which can be attributed to the diminished immunity in older adults and their heightened susceptibility to severe infections. This finding strongly emphasizes the need for enhanced vigilance towards treatment safety in elderly patients. Furthermore, attention should also be directed towards individuals under the age of 20 (3.81%) as a few numbers of AEs continue to be reported. The majority of AEs related to IMI/CIL were reported by pharmacist (55.24%), as well as other health-professional (27.82%) and physician (10.41%), who are considered more reliable sources of reports. There were relatively few AEs reported by consumers (6.22%), suggesting that healthcare workers may be more attentive to IMI/CIL-related AEs during treatment, leading to an increase in this proportion. It is worth noting that despite being among the first drugs to be approved globally by the FDA, the vast majority of reports originated from China (68.53%), indicating unexpected regional differences in reporting trends. This may be attributed to the rapid admission and anti-infection treatment of patients in medical institutions in China, and the large treatment base has led to a significant increase in the number of AE reports. Additionally, with regards to AE outcomes, the majority of cases involved serious events such as other serious (60.14%), hospitalization (24.95%), life threatening (6.34%), death (6.27%) and disability (1.88%). Analysis of the time to onset revealed that AEs predominantly occurred within the first 3 days of drug use, followed by 3 days to 1 week, 1 week to 2 weeks, and very few reported beyond 2 weeks. These findings align with those of Simon Portsmouth et al. (2018). However, it is important to note that there were still some AEs reported after 2 weeks of drug use, indicating potential unforeseen safety risks associated with long-term use of IMI/CIL. These results underscore the significance of early recognition and dynamic monitoring for IMI/CIL-related AEs. Furthermore, there has been an increasing trend in the number of reports on IMI/CIL-related AEs over the years, which may be attributed to either increased drug usage or heightened enthusiasm among medical professionals in reporting such AEs. The aforementioned findings highlight both widespread utilization and potentially severe consequences associated with IMI/CIL and emphasize the necessity for further comprehensive investigations aimed at identifying potential causal relationships in order to advance our understanding of IMI/CIL safety.

The IMI/CIL compound is generally well tolerated as a potent anti-infective agent. Employing the four signal detection methods, the top five SOC were nervous system disorders, psychiatric disorders, general disorders and administration site conditions, investigations and infections and infestations. At the nervous system/psychiatric disorders level, in addition to mental disorder, illusion, epilepsy and encephalopathy documented in the drug description, we have also identified novel signals including delirium, altered state of consciousness, language disorder, disorganized speech, irregular sleep wake rhythm disorder and delirium. Delirium and epilepsy accounted for the largest number of these. The occurrence of IMI/CIL related epilepsy has been widely reported, however, the incidence reported in clinical trials is notably low, ranging from 1.5% to 3% (Verwaest and Belgian Multicenter Study Group, 2000; Calandra et al., 1985; Calandra et al., 1986; Calandra et al., 1988b). In an observational study of 1,754 patients treated with IMI/CIL (Calandra et al., 1988b), seizures were observed in 52 patients (3%), out of which 16 cases (0.9%) were deemed by the investigators to have a strong association with the utilization of IMI/CIL (Calandra et al., 1988b). The study assessed risk factors for IMI/CIL-associated seizures, including underlying central nervous system disorders, renal insufficiency, overdose, and *Pseudomonas aeruginosa* infection (Calandra et al., 1988b). Another study has also suggested that pre-existing cerebral vascular disease may promote seizures prior to drug use, and the blood-brain barrier’s increased vulnerability leads to higher imipenem concentrations in the brain, further exacerbating seizures (Lane et al., 1996). The occurrence of IMI/CIL related nervous system/psychiatric disorders may be attributed to the neurotoxic potential of carbapenem drugs, with an increased risk observed when the dosage is escalated. The toxicity observed is believed to be attributed to the interaction between gamma-aminobutyric acid (GABA) and the drug. Due to its structural similarity with the β-lactam ring, gamma-aminobutyric acid A GABA receptors are particularly susceptible to antagonism by this class of drugs, resulting in a reduction in GABA transmission. Consequently, a range of psychiatric symptoms such as delirium and hallucinations ensue (Norrby, 1996; Teng and Frei, 2022; Grill and Maganti, 2011; Chow et al., 2005). However, the exact cause of this toxicity, whether it is attributed to the drug itself or its metabolites, remains unclear and necessitates further investigation. The study conducted by Koppel et al. observed a higher prevalence of neurological reactions to IMI/CIL in comparison to other carbapenems, such as meropenem and ertapenem (Koppel et al., 2001). This could potentially be attributed to the stronger affinity of IMI/CIL for GABA-agonizing receptors (Miller et al., 2011; Kamei et al., 1983). The change in seizure incidence is attributed to the alkalinity of the side chain attached to the second carbon atom. The higher the alkalinity of the side chain, the greater its affinity for GABA agonist receptors and consequently, the stronger its neurotoxic effect (Chow et al., 2005; Koppel et al., 2001). However, IMI/CIL exhibits a more alkaline side chain compared to other drugs in the same class, which may explain its heightened neurotoxicity (Grill and Maganti, 2008; Snavely and Hodges, 1984). In conclusion, it is imperative for clinicians to conduct further research on the optimal therapeutic window of drug administration in order to mitigate potential neurotoxicity resulting from elevated intracranial drug concentrations. Physicians should refrain from blindly pursuing the desired therapeutic effects through high dosages, while disregarding the associated risks posed by escalating drug concentrations, particularly among patients with pre-existing central nervous system disorders, systemic vascular inflammation, and renal insufficiency.

Most AEs in infection and infestation may be attributed to disease progression in infected patients rather than being specific to the treatment with TZP. At the level of Infections and infestations, antibiotic resistance emerged as the most prevalent AE. Antibiotic resistance has always posed an insurmountable barrier in the treatment of infections. With the rise of drug-resistant bacteria, there is a continuous increase in carbapenem-resistant bacteria. The drug insert does not explicitly mention *acinetobacter* infection, *klebsiella* infection, abdominal infection, and enterococcal infection. However, these conditions should be considered as indications for IMI/CIL rather than being attributed to drug-induced AEs. Therefore, data mining can only serve as a process for discovering suspicious signals, and the causal relationship between drugs and AEs in the database needs to be rigorously evaluated through a signal evaluation process that includes medical assessment. The notable signals observed included pseudomembranous colitis and *Clostridium difficile* colitis. The incidence of *Clostridium difficile* colitis in our study was comparable to that reported by Portsmouth et al. (2018). The occurrence of *clostridium difficile* infection associated with imipenem has been sporadically documented in case reports. In one instance, a geriatric patient with chronic kidney disease undergoing dialysis experienced diarrhea while receiving IMI/CIL treatment for 10 days. Subsequent stool culture confirmed the presence of *Clostridium difficile*, and symptoms resolved upon discontinuation of IMI/CIL therapy and initiation of vancomycin. Investigations also revealed positive signals for fungal test positive and *clostridium* test positive, indicating opportunistic infection. However, the specific mechanism of this infection remains unclear and may be associated with antibiotic-induced disruption of normal intestinal flora, overgrowth of *Clostridium difficile*, and bacterial toxin-mediated damage to enterocyte cytoskeleton integrity (Hassoun, 2018). Additionally, we have also identified several previously unreported AEs, including halo vision, ototoxicity, dysbiosis, foaming at mouth and irregular sleep-wake rhythm disorder. These could be attributed to a direct toxic or allergic reaction to the medication, or it may stem from the patient’s underlying inherent disease or concurrent medication. Ocular toxicity and ototoxicity warrant careful monitoring by healthcare professionals. Considering their potential impact on vision and hearing, as well as the possible detrimental effect on patients’ prognosis, no clinical trials or case reports have been documented thus far. The presence of eye disorders such as halo vision, gaze palsy, and pupillary reflex impairment may be attributed to GABA antagonism, as it has been postulated that a decrease in GABA transmission could lead to heightened excitability in the visual cortex (Kasteleijn-Nolst Trenité et al., 2001).

This study conducted a comprehensive assessment of the safety of IMI/CIL based on the FAERS database; however, the potential inherent limitations of the system itself should not be overlooked. Firstly, as an open-access spontaneous reporting system, FAERS lacks fixed standards and mandatory requirements for data completeness during submission, leading to a significant amount of missing data and confounding factors (Alatawi and Hansen, 2017). Secondly, the absence of a true total number of patients makes it challenging to calculate the true incidence. It is important to note that this study represents only preliminary speculation on drug-related AEs and their potential mechanisms; thus, establishing causal relationships between ADRs is not feasible. ADRs are influenced by various factors such as disease, internal environment, external influences, and individual differences. Therefore, further clinical and basic research is necessary to elucidate these causal relationships. Nonetheless, disproportion analysis serves as an effective method for signal strength mining and provides valuable insight into quantifying risk or causal role. Prospective studies are still required to determine the causality of ADRs with drugs in order to fully comprehend the risks associated with IMI/CIL use. At the same time, medical professionals should continue closely monitoring the occurrence of AEs in clinical practice and implementing timely intervention measures. Despite the constraints of realistic conditions, the analysis of IMI/CIL in this study can still offer favorable perspectives for enhancing drug safety and further research.

## 5 Conclusion

This study assessed the potential correlation between IMI/CIL and ADRs using real-world data from the FAERS database. Through a systematic analysis, previously unreported ADRs as well as known ADRs, including life-threatening serious AEs, were identified. Furthermore, their potential mechanisms were preliminarily explained. Therefore, it is imperative to remain vigilant towards AEs associated with IMI/CIL in clinical practice. However, meticulous attention is requisite to the inherent limitations of the FAERS database and the potential for bias and error. In conclusion, the findings of this study offer comprehensive evidence regarding the safety of IMI/CIL post-marketing, which is valuable for informing future research endeavors.

## Data Availability

The original contributions presented in the study are included in the article/Supplementary Material, further inquiries can be directed to the corresponding authors.
